# Unveiling DNA methylation: early diagnosis, risk assessment, and therapy for endometrial cancer

**DOI:** 10.3389/fonc.2024.1455255

**Published:** 2025-01-20

**Authors:** Minzhen Li, Zhili Xia, Ruiyu Wang, Mingrong Xi, Minmin Hou

**Affiliations:** ^1^ Department of Gynecology and Obstetrics, West China Second University Hospital, Sichuan University, Chengdu, China; ^2^ Key Laboratory of Birth Defects and Related Diseases of Women and Children (Sichuan University), Ministry of Education, Chengdu, China; ^3^ The First Clinical Medical College, Lanzhou University, Lanzhou, China

**Keywords:** DNA methylation, endometrial cancer, early diagnostic biomarker, risk assessment, fertility preservation, prognosis, therapy resistance

## Abstract

Endometrial cancer (EC), one of the most common gynecologic malignancies worldwide, poses a significant burden particularly among young women, with poor treatment outcomes and prognosis for advanced and recurrent patients. Epigenetic changes, encompassing DNA methylation, are involved in the occurrence and progression of tumors and hold promise as effective tools for screening, early diagnosis, treatment strategy, efficacy evaluation, and prognosis analysis. This review provides a comprehensive summary of DNA methylation-based early diagnostic biomarkers in EC, with a focus on recent valuable research findings published in the past two years. The discussion is organized according to sample sources, including cervical scraping, vaginal fluid, urine, blood, and tissue. Additionally, we outline the role of DNA methylation in EC risk assessment, such as carcinogenesis risk, feasibility of fertility preservation approaches, and overall prognosis, aiming to provide personalized treatment decisions for patients. Finally, we review researches on DNA methylation in resistance to first-line treatment of EC and the development of new drugs, and envision the future applications of DNA methylation in EC.

## 1 Introduction

According to the latest cancer statistics spanning 185 countries in 2022, cervical cancer ranked fourth among all women’s cancers with an incidence rate of 3.3%, while endometrial cancer (EC) ranked sixth at 2.1%, making them prominent gynecological tumors globally. In 2022, there were 420,242 newly diagnosed cases of EC and 97,704 deaths (1). EC predominantly affects postmenopausal women, yet 2% to 14% of cases occur in patients with reproductive age, with a notable 7.8% affecting individuals younger than 40 years old (2, 3). Although most patients are diagnosed at an early stage (I-II) confined to the uterus, 10-20% of them experience relapse, with poorer prognosis observed in advanced and recurrent patients (4). Traditional Bokhman’s dualistic model divides EC into type I and type II. Type I tumors, such as endometrioid adenocarcinoma, are generally associated with elevated estrogen levels, accounting for more than 85% of EC cases, and typically exhibit a more favorable prognosis. Conversely, type II tumors, representing about 10% of ECs, are hormone receptor-negative and include serous and clear cell carcinoma, characterized by regrettable outcomes and recurrence rates exceeding 50% (5, 6). Advances in molecular biology and sequencing technology have led to a more nuanced understanding of the molecular mechanisms underlying EC. In 2013, The Cancer Genome Atlas (TCGA) database classified EC into ultramutated/*POLE*mut, hypermutated/MMRd, copy number-high/p53abn and copy number-low/no specific molecular profile (NSMP) types (7, 8). Among these, *POLE*mut and p53abn types show the best and worst prognoses, respectively. TCGA molecular typing provides key information for clinicians to evaluate disease prognosis and formulate individualized treatment, holding promising application prospects. An updated staging system (9) for EC was published by International Federation of Gynecology and Obstetrics (FIGO) in 2023, which adopted molecular classification as one of the staging criteria. Further understanding of the molecular characteristics and regulatory mechanisms of EC is imperative for clinical risk stratification and treatment decisions.

Gene expression is regulated through diverse mechanisms, including gene copy number variations, point mutations, and epigenetic modifications, each playing a critical role in cellular function and tumorigenesis (6). In recent years, increasing attention has been directed towards the pivotal role of epigenetic processes in cancer. DNA methylation is a type of epigenetic modification, and its abnormal alteration stands as a hallmark of human cancer occurrence and development (10). In eukaryotes, the fifth carbon atom of cytosine in the cytidine-phosphateguanosine (CpG) of the genome covalently binds to active methyl group (-CH3) to form 5-methylcytosine (5mC) under the catalysis of DNA methyltransferases (DNMTs), causing epigenetic changes that regulate gene expression (5). CpG is highly aggregated into CpG islands, 70% of which are present in gene promoter regions. Under normal circumstances, about 70% to 80% of CpG outside the CpG islands is methylated, while the vast majority inside CpG islands is hypomethylated or unmethylated (11). DNA methylation process is mediated by DNMTs, which consist of maintenance methyltransferase (DNMT1), and *de novo* DNA methyltransferases (DNMT3A, DNMT3B, and DNMT3L). DNMT1 is required to maintain DNA methylation patterns during cell activity and silence tumor suppressor genes aberrantly in tumor cells. Importantly, DNMT1 is the key enzyme that ensures the smooth transmission of epigenetic marks to the next generation (12). DNMT3A/B are thought to play an important role in shaping the epigenetic landscape of developing individuals, enabling the establishment of new DNA methylation patterns based on environmental factors during embryonic development (13). DNMT3L, a novel regulatory protein for *de novo* methylation, is involved in mediating the activity of DNMT3A/B (14). Dysregulation of DNMTs expression and function has been observed in a variety of diseases, including tumors.

Researches have revealed distinct methylation states at different stages of menstruation cycle. For example, during the proliferative phase, stromal and glandular epithelial cells exhibit higher levels of cytosine methylation, which notably decline in adenocytes during secretory phase (15). These fluctuations may be attributed to the changing steroid hormone levels regulating DNMTs expression and function throughout the menstrual cycle, although the precise molecular mechanisms remain unclear. Compared to normal cells, cancer genomes are characterized by gene-specific hypermethylation of CpG islands and global hypomethylation (16). The methylation of CpG island promoters typically represses gene transcription by inhibiting promoter activity, whereas hypomethylated regions may enhance transcription by facilitating physical interactions between regulatory elements and gene promoters (17, 18). Methylation status in EC is influenced by race (19), with black women displaying lower and more variable DNA methylation levels than white patients. Additionally, methylation patterns correlate with age, aging, body mass index, physical activity, and histological subtype to some extent (20–24). As early as over 20 years ago, studies indicated the hypermethylation of *MLH1* and *PTEN*, leading to tumorigenesis and advanced stage in EC (25, 26). Subsequently, the methylation status of tumor suppressor genes like *RASSF1A*, *APC*, p16, *E-cadherin*, *CDH13*, *ESR1*, and *PRs* have been documented (6, 11). However, there are relatively few reports of hypomethylation of oncogenes, including *PARP1*, *BMP*, *CTCFL*, *PAX2*, *NCAPH*, *MCM*, and *CASP8*. These differentially methylated regions and genes are implicated in many crucial cancer-related biology processes, encompassing cell differentiation, adhesion, invasion, apoptosis, cell cycle control, DNA mismatch repair, and epithelial-mesenchymal transition (EMT) process, cAMP signaling, Wnt signaling, and fibroblast growth factor signaling pathway (27–32). While foundational studies provided valuable insights into the molecular underpinnings of EC, their scope was constrained by methodological limitations and insufficient technological advancements. With the advent of high-throughput technologies, such as whole-genome bisulfite sequencing (WGBS), DNA methylation research has been further expanded. In addition, an increasing number of molecular methylation patterns in EC are being extensively investigated. For instance, novel hypermethylated genes, including *ZSCAN12* and *GYPC*, have been identified as potential diagnostic markers with improved sensitivity and specificity (33).

Apart from DNA methylation, other epigenetic mechanisms, including non-coding RNAs, histone modifications, and chromatin remodeling, also contribute to the pathophysiology of EC. Long non-coding RNAs (lncRNAs) can directly interact with chromatin by forming complexes with DNA, thereby influencing the binding of transcription factor and regulating gene expression (34). MicroRNAs (miRNAs) can pair complementarily with target gene sequences, mediating post-transcriptional suppression of gene expression (35). They play a critical role in the initiation and progression of EC. Histone lactylation promoted the malignant biological behavior of EC cells (36). Histone 3 lysine 27 (H3K27) methylation dysregulation may be an underlying cause of dedifferentiated EC (37), and the histone methyltransferase SMYD3 was highly expressed in EC (38). Chromatin remodeling genes (CRGs), such as *ARID1A*, *CTCF*, and *KMT2D*, were frequently altered in EC and associated with an increased likelihood of lymphovascular and myometrial invasion (39). Additionally, SP-1, ZEB1, and other transcription factors have been widely recognized for their roles in driving the progression of EC (40, 41).

In this review, we summarize the application of DNA methylation in early diagnosis, risk assessment, and therapy of EC, focusing on improving EC diagnostics, treatments, and management strategies.

## 2 DNA methylation-based early diagnostic biomarker candidates in EC

For EC, early detection is paramount because of the high risk of recurrence and poor prognosis in advanced stages. To date, EC diagnosis still relies on the pathology of endometrial biopsy obtained by invasive diagnostic curettage. DNA methylation detection of minimally- and non-invasive specimens is constantly being explored. It has gained wide attention owing to its advantages of easy-to apply and patient-friendly, and is promising to become a new method for tumor screening, diagnosis and prediction. Most tumor biomarker studies have focused on abnormally hypermethylated DNA sequences, possibly because the process of DNA hypomethylation is not fully elucidated in cancer progression and its detection is less sensitive than hypermethylation. Although some literature has summarized DNA methylation-based markers of EC in the past (5, 42, 43), it is relatively incomplete, and many new studies, especially prospective studies, have been published recently.

Abnormally methylated DNA as biomarkers for cancer detection offer the following advantages (16, 44–48): (і) Compared with protein and RNA, DNA methylation modification remains relatively stable after cell destruction, environmental changes and specimen handling, enhancing the probability of successful detection. (ii) Degraded DNA templates can also be utilized for methylation examination, so DNA isolated from exfoliated cells or body fluids is still a suitable sample. (iii) The low quantity of tumor substances in human blood circulation and secretions limited the development of non-invasive detection. PCR amplification of DNA for methylation analysis potentially improves the sensitivity of detection. (iv) Multiple aberrantly methylated DNA can be measured in parallel by different primers, facilitating the establishment of biomarker panels to increase the sensitivity and specificity. (v) DNA methylation occurs early in carcinogenesis process, offering a new option for early cancer detection.

**Figure 1:**
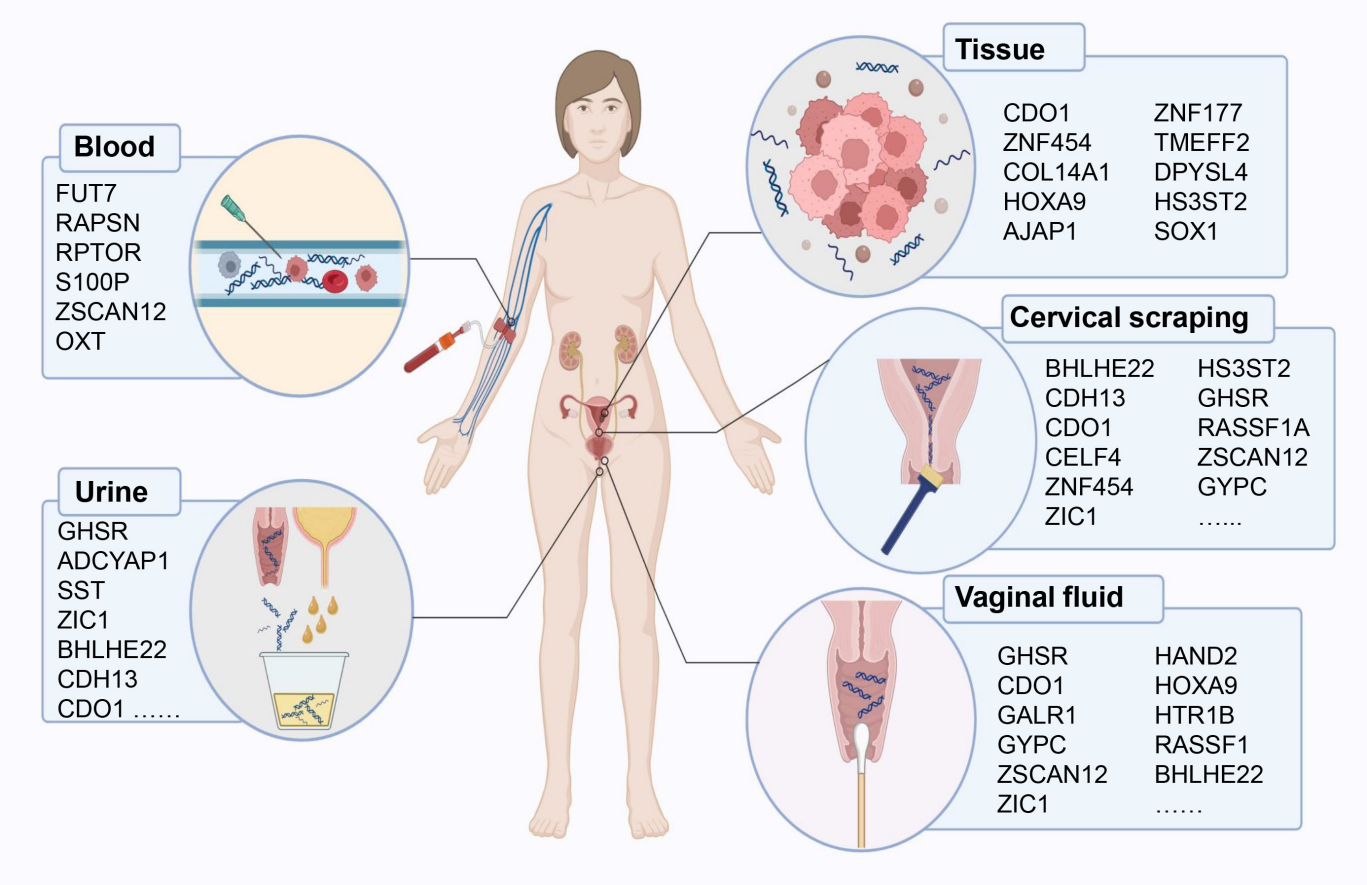
DNA methylation-based early diagnostic biomarkers from various sample sources for endometrial cancer detection.

Here, we review diagnostic biomarkers of DNA methylation published so far, and discussed them separately according to different specimen types (**Figure 1**).

### 2.1 Cervical scrape samples

With the widespread implementation of cervical cancer screening, some asymptomatic ECs have been incidentally detected via cervical cytology. Diseased endometrial cells and/or cell-free DNA in cervical scrape samples provide an opportunity to leverage more sensitive DNA methylation molecular testing.

*BHLHE22*, a member of basic helix loop helix (BHLH) transcription factor family, is highly methylated in EC tissues and involved in regulating the immune microenvironment, which is closely linked to tumor progression (49). Another BHLH family member, *HAND2*, inhibits the ligand-dependent transcriptional activation of estrogen receptor (ER)-α (50) and exhibits a state of hypermethylation and gene silencing in EC (51). Cysteine dioxygenase 1 (*CDO1*), a non-haeme iron dioxygenase, is hypermethylated in EC and related to tumor growth *in vivo (* 52). Tumor suppressor genes such as *RASSF1A* and *CDH13* exhibit high methylation frequencies in EC and play crucial roles in early lesions, suggesting their utility in early diagnostic panels (6). Although genes like *PCDHGB7*, which is hypermethylated in several cancer types (53, 54), have shown potential diagnostic value, their specific relevance to EC requires further validation. Yuan et al. (55) evaluated *PCDHGB7* methylation detection performance in endometrial brush samples (Tao brush) and cervical scrapes (Pap brush), demonstrating its ability to differentiate malignant from normal endometrium with high specificity and sensitivity.

Most studies measured the efficacy of combined detection of multiple methylation biomarkers to achieve greater diagnostic values. A multicenter hospital-based, two-stage validation study (56) established an algorithm model (MPap assay) based on age, BMI, and DNA methylation status of *BHLHE22* and *CDO1* in cervical scrapings for diagnosing stage I EC. This study included a total of 592 women with abnormal uterine bleeding (AUB) and demonstrated that MPap assay outperformed transvaginal sonography (TVS) with a sensitivity value exceeding 92%. Kong et al. collected cervical exfoliated cell samples from 143 postmenopausal women with suspected endometrial lesions and compared the efficacy of DNA methylation testing (*CDO1* and *CELF4*) with or without TVS in screening EC. They found that dual-gene methylation for diagnosis exhibited the highest sensitivity (87.5%) and specificity (90.8%), and TVS combined with methylation tests further improved sensitivity to 100%, albeit not specificity (57). Wever et al. demonstrated that a DNA methylation marker panel by combining *CDH13* + *CDO1* + *ZIC1* gained an increased AUC value of 0.97 and a satisfactory sensitivity of 93% (58). Pap brush samples from EC patients showed significantly elevated methylation levels of *RASSF1A* and *HIST1H4F*, which successfully detected EC with the AUC values of 0.938 and 0.951, respectively (59). A prospective, observational cohort study (Women’s cancer risk IDentification – quantitative polymerase chain reaction test for Endometrial Cancer, WID-qEC test) (60) based on the methylation of *ZSCAN12/GYPC* verified the presence of EC in 137 cervical smear specimens, with a sensitivity of 97.2% and a specificity of 75.8%. WID-qEC test presented similar sensitivity and AUC but increased specificity in comparison to TVS. Interestingly, compared with DNA mutation analysis, WID-qEC test showed similar specificity but significantly increased AUC and sensitivity. Recently, researchers developed a new WID-EC (Women’s cancer risk IDentification – Endometrial Cancer) test (61) based on DNA methylation at 500 CpG sites, which has the advantage of identifying endometrial, cervical, ovarian, and breast cancers from a single cervical liquid-based cytology sample on the same test platform. For EC detection, the AUC of WID-EC test was 0.92 (95% CI: 0.88-0.97), with the sensitivity of 86% and specificity of 90%. Besides, methylation of *POU4F3/MAGI2* (62), *BHLHE22/CDO1/CELF4* (63), *BHLHE22/CDO1* (64) genes has also been proven to play a role in the diagnosis of EC. All the biomarkers are presented in detail in **Table 1**.

**Table 1:** DNA methylation-based biomarker candidates from cervical scrape samples for the diagnosis of endometrial cancer.

Biomarker gene(s)	AUC	Sensitivity (%)	Specificity (%)	Reference
*ADCYAP1*	0.79-0.88	57	91	(58, 65, 66 ^a^)
*ASCL2*	0.76-0.81	U	U	(65, 66 ^a^)
*BHLHE22*	0.84-0.95	73-83.7	85-93.7	(58, 63, 64)
*BHLHE22*, *CDO1*	U	84.8	88	(64)
*BHLHE22*, *CDO1*, *HAND2*	U	87	86	(64)
*BHLHE22*, *CDO1*, *TBX5*	U	89.1	80	(64)
*BHLHE22*, *CDO1*, *CELF4*	U	91.8	95.5	(63)
*CDH13*	0.68-0.86	48	90	(58, 65, 66 ^a^)
*CDH13*, *CDO1*, *ZIC1*	0.97	93	90	(58)
*CDO1*	0.84-0.96	75-87	90.79-94.3	(57, 58, 63, 64, 67 ^b^)
*CDO1*, *CELF4*	0.89	87.5	90.8	(57)
*CDO1*, *ZNF454*	0.931	90.91	86.84	(67 ^b^)
*CELF4*	0.87-0.94	78.6-96	78.7-95.4	(57, 63)
*GALR1*	0.63	42	88	(58)
*GHSR*	0.91	79	93	(58)
*GTF2A1*	0.45	U	U	(65)
*GYPC*, *ZSCAN12*	U	97.2	75.8	(60)
*HAAO*	0.68	U	U	(65)
*HAND2*	0.62-0.77	44	86	(58, 64)
*HIST1H4F*	0.951	U	U	(59)
*HOXA9*	0.58	U	U	(65)
*HS3ST2*	0.75-0.8	U	U	(65, 66 ^a^)
*HSP2A*	0.68	U	U	(65)
*HTR1B*	0.67-0.68	U	U	(65, 66 ^a^)
*MAGI2*	0.9	90	75	(62)
*MME*	0.83-0.86	U	U	(65, 66 ^a^)
Mpap (*BHLHE22*, *CDO1*, Age, BMI)	0.9	92.5	73.8	(56)
*NPY*	0.6-0.76	U	U	(65, 66 ^a^)
*PCDHGB7*	0.83	61.29	95.31	(55)^c^
*PCDHGB7*	0.86	80.65	82.81	(55)^d^
*POU4F3*	0.8	83	69	(62)
*RASSF1*	0.86	U	U	(65)
*RASSF1A*	0.833-0.938	63	96.3	(59, 68)
*SST*	0.74	64	75	(58)
*TBX5*	0.7	U	U	(64)
WID-EC test	0.92	86	90	(61)
*ZIC1*	0.78	79	67	(58)
*ZNF454*	0.905	79.55	93.42	(67 ^b^)
*ZNF662*	0.89	92	80	(63)

a and c: data from endometrial brush samples (Tao brush); b: statistical results derived from the comparison between EC+AH group and BE group; d: data from cervical scrapes (Pap brush); EC, endometrial cancer; AH, atypical hyperplasia; BE, benign endometrium; U, unknown; WID-EC test, Women’s cancer risk IDentification – Endometrial Cancer test.

### 2.2 Vaginal fluid

Cervical cancer screening programs have shifted in many countries from cytology to preliminary testing for human papillomavirus, resulting in a decline in the proportion of asymptomatic ECs detected by cervical scrapes and the increasing dominance of vaginal fluid specimens. Typically, endometrial shed cells are difficult to preserve for at-home self-testing, but vaginal fluid contains unique DNA mutations, sequence alterations or methylation signals of gene promoter that can indicate the presence of endometrial diseases (69, 70). Patient self-collection of vaginal fluid using intravaginal tampon or swab may offer an effective method for early detection at home. In fact, as early as 2004, methylated DNA in tampons was being explored as a potential test for EC (71).

Bakkum-Gamez et al. (65) reported, for the first time, DNA hypermethylation of *HOXA9* in EC and demonstrated the feasibility of methylation of *HTR1B* (AUC 0.82), *RASSF1* (AUC 0.75) and *HOXA9* (AUC 0.74) genes in tampon specimens as markers for EC, consistent with Sangtani’s report (70). Subsequently, they set a 28- methylated DNA marker (MDM) panel, including *MAX.chr12*, *CDH4*, *c17orf64*, *EMX2OS*, *NBPF8* and others, which highly discriminated between EC and benign endometrium (sensitivity: 82%; specificity: 96%; AUC: 0.91) (69). The aforementioned WID-qEC test (33) in 2023 evaluated the real-world performance of *ZSCAN12* and *GYPC* DNA methylation in vaginal tampons for screening EC patients from women with AUB. The results showed that WID-qEC test achieved an AUC of 0.94, a sensitivity of 90.9%, and a specificity of 97.3%, all superior to TVS. Moreover, the WID-qEC study recruited 69 women presenting for postmenopausal bleeding and correctly identified 100% of cases who diagnosed EC, with a specificity of 89.1% (60). For details, see **Table 2**.

**Table 2:** DNA methylation-based biomarker candidates from vaginal fluid samples for endometrial cancer detection.

Biomarker gene(s)	AUC	Sensitivity (%)	Specificity (%)	Reference
*ADCYAP1*	0.67-0.68	47	93	(58, 65)
*ASCL2*	0.69	U	U	(65)
*BHLHE22*	0.76	60	89	(58)
*CDH13*	0.67-0.69	45	93	(58, 65)
*CDO1*	0.91	78	94	(58)
*CDO1*, *GHSR*, *ZIC1*	0.94	89	92	(58)
*GALR1*	0.7-0.93	44-92.7	78.9-95	(48, 58)
*GHSR*	0.85	75	87	(58)
*GTF2A1*	0.55	U	U	(65)
*GYPC*, *ZSCAN12*	0.94	90.9-100	89.1-97.3	(33, 60)
*HAAO*	0.68	U	U	(65)
*HAND2*	0.65-0.97	46	88	(51, 58)
*HOXA9*	0.77	37.8	100	(65, 70)
*HOXA9*, *HTR1B*, *RASSF1*	U	60	100	(70)
*HS3ST2*	0.73	U	U	(65)
*HSP2A*	0.67	U	U	(65)
*HTR1B*	0.81	38.9	100	(65, 70)
*28-MDM panel*	0.91	82	96	(69)
*MME*	0.69	U	U	(65)
*NPY*	0.67	U	U	(65)
*RASSF1*	0.79	40	100	(65, 70)
*SST*	0.62	28	95	(58)
*ZIC1*	0.62	39	85	(58)

*28-MDM panel: MAX.chr12, CDH4, c17orf64, EMX2OS, NBPF8, SFMBT2, JSRP1, DIDO1, MAX.chr10, MPZ, ZNF506, GATA2, VILL, MAX.chr14, CYTH2, LRRC8D, LYPLAL1, MAX.chr8, SQSTM1, ZNF323, OBSCN, ZNF90, LRRC34, GDF7, MDFI, EEF1A2, LRRC41,* and *SEPT9*; MDM: methylated DNA marker.

### 2.3 Urine

Given the anatomical proximity of the urethra to vagina, urine may serve as a valuable specimen for EC testing. Recent research has shown that EC cells shed into vaginal debris through the cervix and can be detected in urine samples (72). Urine samples can be conveniently collected at home, reducing economic costs and healthcare burdens. High levels of DNA methylation are present in urine from EC patients (73). The methylation degree of different urine fractions varied slightly, and full void urine got the highest diagnostic potential for EC compared to urine sediment and supernatant (73). The combined detection of DNA methylation of *CDH13*, *GHSR*, and *SST* in urine yielded an AUC value of 0.95 (95% CI: 0.92-0.98), and increased the sensitivity compared with single gene examination (90% vs. 34%-87%) (58) (**Table 3**).

**Table 3:** DNA methylation-based biomarker candidates from urine samples for endometrial cancer diagnosis.

Biomarker gene(s)	AUC	Sensitivity (%)	Specificity (%)	Reference
*ADCYAP1*	0.83	62	93	(58)
*BHLHE22*	0.85	66	98	(58)
*CDH13*	0.9	80	92	(58)
*CDH13*, *GHSR*, *SST*	0.95	90	90	(58)
*CDO1*	0.9	85	84	(58)
*GALR1*	0.79	63	86	(58)
*GHSR*	0.93-0.95	87	87	(58, 73)
*HAND2*	0.71	47	91	(58)
*SST*	0.61-0.92	34	93	(58, 73)
*ZIC1*	0.78-0.86	54	95	(58, 73)

### 2.4 Blood

Circulating tumor DNA (ctDNA), the small fragments of DNA released into the blood by cancer cells, has been used for continuous monitoring of tumor burden in the clinic because of its accessibility and low trauma. Due to the mutation heterogeneity of EC, previous studies mainly based on ctDNA mutation detection have been hampered, but DNA methylation analysis is expected to overcome this challenge (4, 74, 75). Analyzing methylation information in ctDNA is beneficial for screening, early diagnosis and therapy of cancer, prediction of disease recurrence and response to treatment (76). Researchers have extensively explored DNA methylation-based testing of blood samples for the detection of colorectal (77), ovarian (78), lung (79), liver (80) cancer and even multi-tumor (81, 82). However, there have been limited studies on EC. Schuhn et al. assessed expression levels of 7 MDMs in blood samples from women with EC (20 cases), benign endometrium (BE, 14 cases), and normal control (NC, 157 cases). The AUC of diagnosing ECs from NC group was 0.772 for *RAPSN*_CpG_6 and 0.75 for *S100P*_CpG_2,3. For diagnosing ECs from BE cases, the AUC was 0.773 for *RPTOR*_CpG_2,3 and 0.752 for *FUT7*_CpG_6 *(* 83). Combining the methylation of *ZSCAN12* and oxytocin (*OXT*) in ctDNA accurately classified EC patients from control group with the AUC value of 0.99 (sensitivity: 98%; specificity: 97%) (4) (**Table 4**).

**Table 4:** DNA methylation-based biomarkers from blood samples for endometrial carcinoma detection.

Biomarker gene(s)	AUC	Sensitivity (%)	Specificity (%)	Reference
*FUT7*	0.744-0.752	55.6-66.7	84.6-92.3	(83)^a^
*RAPSN*	0.772	73.7	75.2	(83)^b^
*RPTOR*	0.729-0.773	63.2-94.7	46.2-84.6	(83)^a^
*S100P*	0.75	89.5	54.5	(83)^b^
*ZSCAN12*, *OXT*	0.99	98	97	(4)

a: statistical results derived from the comparison between EC group and BE group; b: results derived from the comparison between EC group and NC group; NC, normal control.

### 2.5 Tissue

DNA methylation testing also provides a potential option for detecting malignancy in endometrial tissue. Lai et al. (84) explored the potential role of MDMs in screening the presence of hidden EC in women with atypical hyperplasia (AH). The 61 AH patients whose endometrium was collected for methylation analysis all underwent hysterectomy within three months. The AUC values of *AJAP1*, *HS3ST2*, and *SOX1* methylation for EC diagnosis were 0.81, 0.72, and 0.70, respectively. Wang et al. (67) categorized EC and AH as the malignant group, and BE and NC as the benign group. It was found that the methylation testing panel of *CDO1*+*ZNF454* could distinguish the two groups well with the AUC of 0.911 (sensitivity: 92.68%; specificity: 82.26%). In addition, promoter methylation of *COL14A1*, *DPYSL4*, *HOXA9*, *TMEFF2*, and *ZNF177* genes was valuable in identifying undetected EC within endometrial hyperplasia (85) (**Table 5**). Compared to other specimens, the process of tissue acquisition is more invasive, so methylation detection in tissue for cancer screening and diagnosis may be more suitable to provide supplementary information in cases with unknown or inconclusive pathologic findings.

**Table 5:** DNA methylation-based biomarkers from tissue samples for endometrial carcinoma diagnosis.

Biomarker gene(s)	AUC	Sensitivity (%)	Specificity (%)	Reference
*AJAP1*	0.81	U	U	(84)
*CDO1*	0.91	80.49	93.55	(67)^a^
*CDO1*, *ZNF454*	0.911	92.68	82.26	(67)^a^
*COL14A1*	0.96	92.3	94.4	(85)
*COL14A1*, *HOXA9*	U	76.9	100	(85)
*COL14A1*, *TMEFF2*	U	61.5	100	(85)
*DPYSL4*	0.63	61.5	94.4	(85)
*HOXA9*	0.81	80.8	94.4	(85)
*HS3ST2*	0.72	U	U	(84)
*SOX1*	0.7	U	U	(84)
*TMEFF2*	0.9	65.4	94.4	(85)
*ZNF177*	0.95	92.3	94.4	(85)
*ZNF177*, *COL14A1*	U	88.5	100	(85)
*ZNF177*, *COL14A1*, *HOXA9*	U	73.1	100	(85)
*ZNF177*, *HOXA9*	U	76.9	100	(85)
*ZNF177*, *TMEFF2*	U	61.5	100	(85)
*ZNF454*	0.838	78.05	82.26	(67)^a^

a: statistical results derived from the comparison between EC+AH group and BE+NC group.

Currently, no mature methylation detection kit has been approved for clinical practice of EC, but a series of clinical trials, both domestically and internationally, are underway to achieve early diagnosis and therapy of tumor. For example, the ongoing clinical trials at Peking Union Medical College Hospital aspire to validate the accuracy of host DNA methylation of *CDO1*+*CELF4* (NCT05290922) in cervical cytology samples and *BHLHE22*+*CELF4*+*HAND2*+*ZNF177* (NCT04651738) in peripheral serum for EC screening.

Despite significant variability in the diagnostic efficacy of these candidate genes, it is believed that with larger cohort studies, further subpopulation analysis, and the exploration of more promising markers, methylation testing for EC could represent a landmark advancement in tumor screening, akin to colorectal cancer. Relying solely on DNA methylation markers may not fully capture the complex molecular characteristics of the disease. Integrating methylation data with other omics datasets, such as transcriptomics and proteomics, not only deepens our understanding of the molecular mechanisms underlying EC but also aids in the development of more comprehensive diagnostic panels. Furthermore, this multi-omics approach better addresses the challenges posed by heterogeneity of EC.

## 3 DNA methylation in risk assessment of EC

### 3.1 Carcinogenesis risk

The vast majority of patients with EC have prodromal symptoms such as AUB, but only 5-10% of women with postmenopausal AUB have an underlying malignancy (86). Repeated intrauterine manipulation and endometrial biopsy increase the psychological burden in women and generate waste of social medical resources. A predictive scoring system is needed to distinguish women at low or high risk of tumorigenesis, enabling personalized intervention and treatment. Researchers performed DNA methylation analysis in benign tissues from 23 women who later developed EC within a median interval of 1 year. The results showed an increased methylation trend in all of these endometrial tissues, and the AUC for *ADCYAP1* and *HAND2* methylation to predict the risk of future EC were 0.71 and 0.83, respectively (87). The WID-qEC test, based on *ZSCAN12* and *GYPC* DNA methylation, appeared to be an excellent predictor of near-term (<1 year) EC risk (sensitivity: 91%; specificity: 100%), but not effective in assessing long-term (≥ 1 year) cancer risk (sensitivity: 20%) (60). As part of routine cervical screening in 150 healthy women, the WID-EC test successfully predicted more than half of cases developing EC within 3 years (AUC: 0.82; sensitivity: 52%; specificity: 98%) (61). The methylation status of *HS3ST2* and *KLF4* was detected in cancerous, hyperplastic and normal endometrial tissues, and multinomial logistic regression analysis showed that *HS3ST2* and *KLF4* were predictors of endometrial hyperplasia and carcinoma. Notably, the AUC value of *KLF4* methylation in predicting EC was 0.95 (88).

### 3.2 Feasibility of fertility preservation

In recent years, the incidence of EC in young women has been on the rise. For young women diagnosed with grade 1, early-stage endometrial adenocarcinoma, fertility preservation therapy is considered viable, with remission rates ranging from 76.2% to 81.4%, but there is still a recurrence rate of 24-40% after complete remission (89). For fertility preservation, it is important to screen people with a truly low risk of relapse through molecular biology and epigenetics. Through genome-wide DNA methylation analysis in 49 fresh-frozen and 31 formalin-fixed paraffin-embedded tissue samples, Hirano et al. (90) elucidated that differently methylated genes accumulated in pathways associated with fibroblast growth factor and nuclear factor-κB signaling portended a higher risk of recurrence in early-onset EC (aged ≤40 years). Using up to 6 of 8 CpG sites for *LPP*, *FOXO1*, *RNF4*, *EXOC6B*, *CCPG1*, *RREB1* and *ZBTB38* as DNA methylation diagnostic criteria could effectively assess recurrence risk (sensitivity and specificity greater than 91.3%), providing insights into the safety of fertility preservation therapy for patients with early-onset EC (90). Another study revealed that a cluster with less aggressive clinicopathological features in early-onset EC patients (34 samples), characterized by changes in DNA methylation levels of 18 genes, including *HOXA9*, *HOXD10* and *SOX11 (* 28). These results suggested that DNA methylation analysis might help predict EC patients which were less risky and suitable for fertility preservation therapy. However, these findings are based on observational studies and require further functional and longitudinal validation. The limited number of existing studies emphasizes the need for larger, multi-center investigations to confirm the contribution of these methylation markers in predicting the feasibility of fertility preservation for EC patients.

### 3.3 Prognosis

DNA methylation is more suitable for cancer prognosis assessment than gene expression because of its data stability (91). Although some researchers suggested that *MLH1* methylation cannot predict OS or disease-free survival (DFS) (92), it is widely accepted that EC patients with hypermethylation of *MLH1* promoter have a worse prognosis and relapse more frequently, even in women traditionally considered to be at low risk for recurrence (24, 93, 94). Pasanen et al. (95) proved *MLH1* methylated patients appeared to get poor disease-specific survivals than MMR proficient ones. Shikama et al. (96) found significant difference in OS between deficient MMR (dMMR) nonmethylated and sporadic cases, that is, patients with intact MMR protein expression or hypermethylated *MLH1*. Moreover, in the subset receiving adjuvant therapy, dMMR nonmethylated patients showed a more favorable trend in DFS. In advanced endometrioid adenocarcinoma, *MLH1*-methylated group was more likely to relapse than the MMR-proficient group, despite receiving similar adjuvant therapy (97). However, a single institution retrospective research (98) found that whole pelvic radiotherapy and radiotherapy combined with chemotherapy correlated with poorer prognosis in dMMR nonmethylated but not in *MLH1* methylated cases, suggesting that *MLH1* methylated EC patients may be relatively sensitive to adjuvant therapy. Therefore, analyzing *MLH1* methylation status helps to identify a subgroup of patients with poor survival, with a view to providing more proactive and individualized treatment decisions. But the relationship between *MLH1* methylation and the effectiveness of adjuvant therapy needs to be considered, because of intricate influencing factors and current inconclusive findings.

Downregulation of E-cadherin is an important part of EMT process, which represents the loss of cell-cell adhesion structures and the increased cell motility and invasion (99). By analyzing the methylation status in 142 endometrial tissues, Saito et al. suggested that E-cadherin gene methylation occurred in association with the tumor invasion ability such as tumor dedifferention, myometrial invasion and lymph node metastasis in EC (100). *TESTIN* hypermethylation was significantly associated with deep myometrial invasion and lymph node metastasis, and may lead to unfavorable treatment outcomes by enhancing the EMT process in EC (32). Epigenetic silencing of *PTEN* was linked to advanced stage, microsatellite instability, and poor prognosis (26). *TBX2*, *CHST11* and *NID2* were also related to unfavorable clinical predictive and prognostic factors in EC (101).

Most other studies of methylated molecules associated with prognosis are based on bioinformatics. Li et al. screened five differentially methylated genes (*GBP4*, *OR8K3*, *GABRA2*, *RIPPLY2*, and *TRBV5-7*) associated with prognosis of EC from TCGA database and established a risk score model, which predicted 5-year survival with an AUC of 0.926 (102). The gene co-methylation network was constructed based on the DNA methylation data of EC in TCGA, identifying a series of prognostic related markers, including *AURKA*, *CHTF18*, *EZH2*, *FBXW7*, *JAG1*, etc., and molecular typing of EC was performed (91). The methylation of *ELFN1-AS1* and *ZNF132* could classify EC patients into high-risk and low-risk prognostic groups (103). Methylation of 12 genes, including *MESDC1*, *LRRTM1*, *NOVA1*, *C5orf38* and *IRX2*, which were enriched in MAPK signaling, hippo signaling, oxytocin signaling and cell adhesion molecule pathways, had been shown to be correlated with the prognosis of patients with EC (44). Patients with hypermethylation of *CDC20* and *CCNA2* showed longer overall survival (104). *SYTL1* DNA methylation was inversely correlated with OS in EC (105). In addition, some studies have also hypothesized that immune infiltration may be part of the prognostic influence of certain methylated genes, such as *PARVG/SYNE4/CDO1 (* 106) and *CIRBP/INPP5K (* 107). Despite many attempts at the prognostic value of DNA methylation in bioinformatics, further validation is needed.

## 4 DNA methylation in EC therapy

### 4.1 Therapy resistance

Endocrine therapy, particularly high-dose progesterone, has been widely used in ER/progesterone receptor (PR)-expressing early-stage EC patients, but progesterone resistance still occurs in some cases. Moreover, the response rates are poor in advanced and recurrent women. Repeated use of progesterone may lead to loss of PR and eventually treatment resistance (108). In addition, some epigenetic mechanisms and signaling pathways are also involved in the formation of progesterone insensitivity. CpG islands exist in the promoter region and first exon of PR gene, where DNA methylation is abnormally active in EC, endometriosis, breast cancer and other hormone-related diseases. Studies have shown that aberrant DNA methylation brings about suppressed or even absent expression of PR gene in tumors (108, 109). PRA and PRB, two isoforms of PR, play different roles in progesterone therapy. PRB works by activating gene transcription, while PRA interferes with the therapeutic effect via inhibiting PRB function. Interestingly, *PRA* and *PRB* are not methylated simultaneously, suggesting independent regulation of *PRA* and *PRB* methylation processes (110, 111). In breast cancer, low PR levels were significantly associated with poor prognosis, and the methylation of *PRA*, rather than *PRB*, worsened tamoxifen treatment outcomes (112). More than a decade ago, it was demonstrated that DNMT inhibitors (DNMTi) combined with endocrine therapy were effective against constitutive-resistant breast carcinoma with high DNMT levels (113). In EC, PR gene methylation was related to congenital progesterone resistance (114). A significantly higher frequency of PR promoter methylation was observed in metastatic tumor than in primary lesion (115), posing a barrier to hormone therapy in advanced metastatic cases. Small molecule DNMTi sensitized poorly differentiated PRB-negative EC to progesterone therapy (116). Moreover, Jones et al. observed that *HAND2* methylation levels were significantly higher in non-cancerous hyperplastic endometrium that did not respond to progesterone for 3 months compared with endometrial lesions that subsided after treatment, suggesting that progesterone responsiveness was reliant upon *HAND2* expression (51).

**Figure 2:**
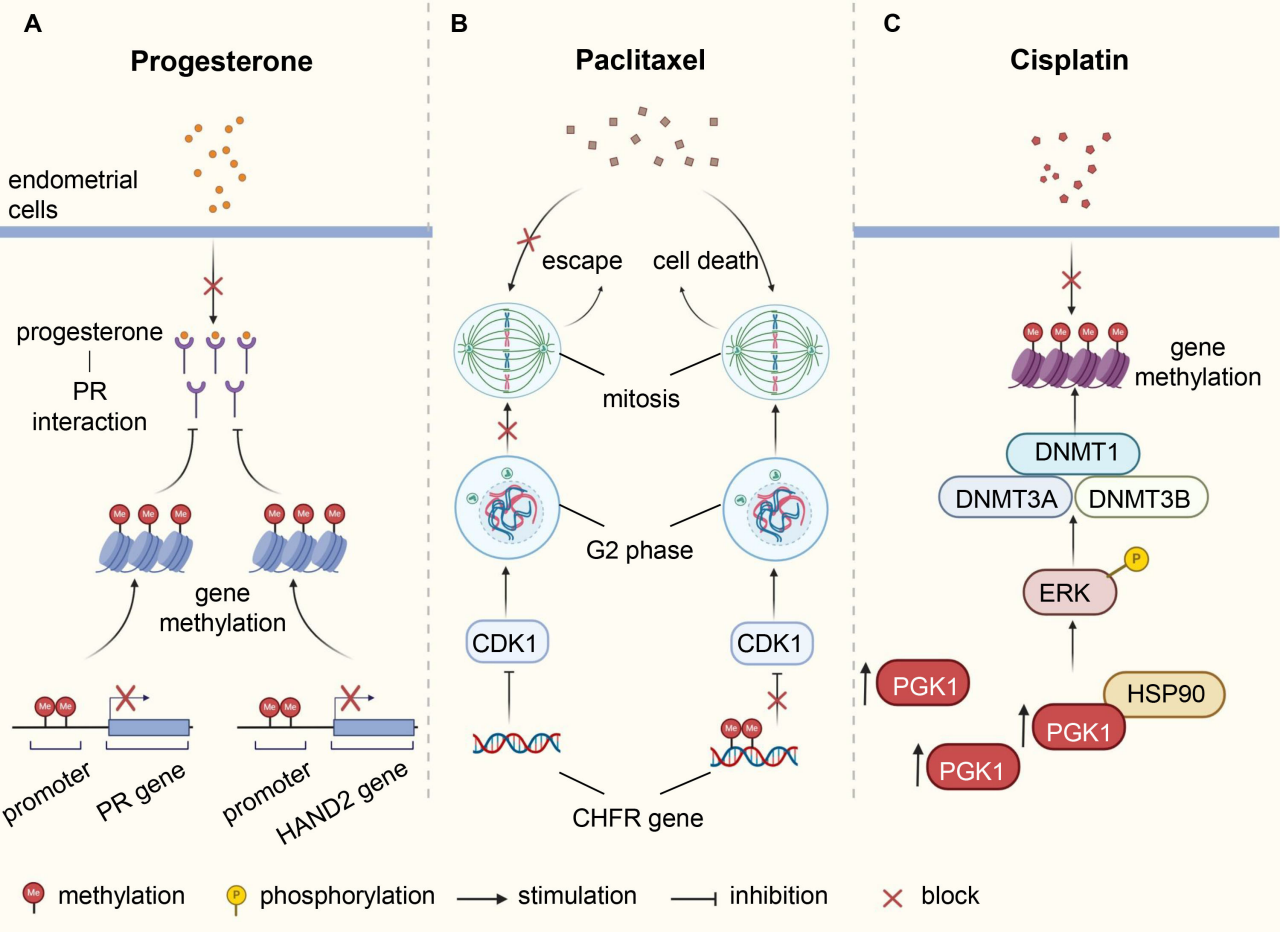
Underlying mechanisms of DNA methylation involved in therapy resistance of endometrial cancer. **(A)** Abnormal methylation of *PR* and *HAND2* leads to downregulation of their protein expression, affecting the interaction between progesterone and PR, resulting in resistance of endometrial cells to progesterone. **(B)** The CHFR protein delays cell entry into mitosis by inhibiting CDK1, thereby weakening the cytotoxic effects of paclitaxel. Methylation of *CHFR* gene in EC cells leads to protein inactivation, heightening sensitivity to paclitaxel. **(C)** Elevated levels of PGK1 in EC induce ERK phosphorylation by directly binding to HSP90, causing upregulation of DNMTs expression, increasing methylation levels, and enhancing resistance to cisplatin. PR, progesterone receptor; EC, endometrial cancer; CHFR, checkpoint with forkhead-associated and ring finger; CDK1, cyclin dependent kinase 1; PGK1, phosphoglycerate kinase 1; DNMTs, DNA methyltransferases.

Chemotherapy is one of the main treatment strategies for post-surgery patients and those with advanced or recurrent EC. Commonly used chemotherapy drugs include paclitaxel, platinum, doxorubicin, and topotecan. Drug resistance, significantly reducing progression-free survival (PFS) and overall survival (OS), is related to gene mutation, microenvironment change, abnormal regulation of signaling pathways and other factors, covering variants in DNA methylation patterns (14). The checkpoint with forkhead-associated and ring finger (CHFR) protein acts as a cell cycle checkpoint component to delay the entry of cells into mitosis by diminishing cyclin dependent kinase 1 (CDK1) activity. When tubulin homeostasia is disrupted by agents such as paclitaxel, CHFR-positive cells stall in the G2 phase and even retreat from early mitosis, allowing them to escape, to some extent, cell death (117). The *CHFR* gene is often inactivated by methylation in cancer cells, and its methylation is associated with poor prognosis and increased sensitivity to paclitaxel in multiple cancer types, including ovarian and gastric cancers (117, 118). In paclitaxel-sensitive EC cells, *CHFR* gene hypermethylation occurred more frequently, and restoring CHFR expression could reduce cell sensitivity to paclitaxel (119, 120). Zhou et al. showed that phosphoglycerate kinase 1 (PGK1) mediated upregulation of DNMTs (DNMT1, DNMT3A and DNMT3B) through the HSP90/ERK pathway, leading to increased methylation levels and enhancing cisplatin resistance in EC (121) (**Figure 2**). Fialkova et al. examined the changes of promoter DNA methylation in apoptosis-associated genes and observed the influences of *BCL2L11*, *CIDEB* and *GADD45A* methylation in endometrial carcinogenesis, which may contribute to deregulation of mitochondrial apoptotic pathway and development of pro-apoptotic drug resistance (122).

### 4.2 New drugs

DNA methylation is reversible and dynamic. Under normal circumstances, DNA methylation and demethylation are in dynamic equilibrium, with extensive changes in DNA methylation patterns observed at all stages of tumors (123). This indicates that DNA demethylating agents may hold promise in reprogramming tumor cells back to normal state, representing a new strategy of cancer therapy.

DNA demethylating compounds are generally referred to as DNMTi, which can be divided into two groups: nucleoside and non-nucleoside inhibitors (124). Nucleoside analogues have been developed for over 40 years since their DNA demethylation activity was first discovered in 1980 (125). They capture DNMTs by binding DNA for proteasomal degradation. As representative drugs, azacitidine and decitabine have been approved by the US Food and Drug Administration (FDA) against hematological malignancies, including myelodysplastic syndrome (MDS) and acute myeloid leukaemia (AML) (126). Decitabine, also known as 5-aza-2’-deoxycytidine, is the most common medicament used to induce DNA demethylation. Treatment of EC cells with decitabine resulted in the downregulation of DNMT3B and upregulation of *MLH1*. This was accompanied by cell growth inhibition, cycle arrest and apoptosis, along with elevated E-cadherin and decreased Bcl-2 expression (127, 128). Azacitidine appeared to target DNMTs more effectively than decitabine in *MLH1*-hypermethylated mismatch repair (MMR) deficient ECs (129). Azacitidine could rescue secreted protein acidic and rich in cysteine (SPARC), an albumin-binding protein, from a hypermethylated state, thereby increasing albumin-bound paclitaxel accumulation at EC lesions (130). Guadecitabine (SGI-110) is a second-generation demethylation drug that prolongs plasma half-life and improves binding strength compared to azacitidine and decitabine due to its resistance to degradation by cytidine deaminase (131). Zebularine, a relatively new cytidine analogue, is more stable and has a longer half-life than azacitidine (132). Unfortunately, there is no research evidence available for these drugs in EC.

**Figure 3:**
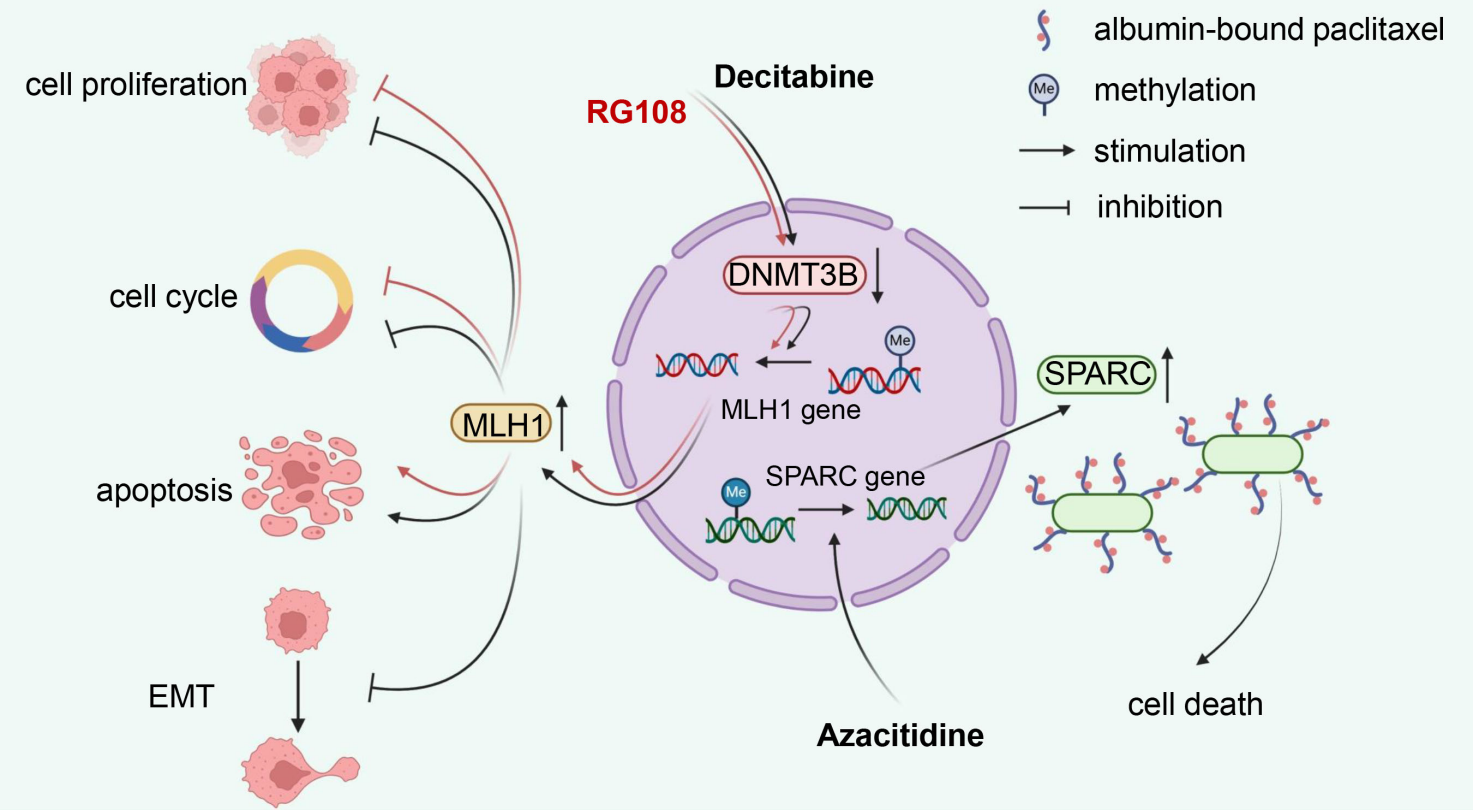
Mechanism illustration of demethylation drugs in endometrial cancer. Nucleoside analog decitabine and non-nucleoside inhibitor RG108 reduce *MLH1* methylation levels by inhibiting DNMT3B, thereby inhibiting proliferation, inducing cell cycle arrest, and apoptosis in EC cells. Additionally, decitabine can suppress the EMT process. Another nucleoside drug, azacitidine, promotes the demethylation process of SPARC, an albumin-binding protein, which enhances the accumulation of albumin-bound paclitaxel in EC lesions, consequently increasing its anti-tumor efficacy. DNMT3B, DNA methyltransferase 3B; EMT, epithelial-mesenchymal transition; SPARC, secreted protein acidic and rich in cysteine; EC, endometrial cancer.

Nucleoside analogues binding to DNA may induce mutagenic damage that results in unnecessary toxicity and side effects, therefore, non-nucleoside analogues are currently under extensive investigation. Unlike nucleoside analogues, non-nucleoside inhibitors do not mimic cytosine. Instead, they work by directly binding and inhibiting specific target proteins (132). In EC, RG108, suppressing DNMT3B and upregulating *MLH1*, could inhibit tumor cell growth, block cell cycle, and induce apoptosis, seeming to be considered as a new candidate drug for EC treatment (133). However, other non-nucleoside inhibitors, such as SGI-1027, hydralazine, procainamide and EGCG (134), have not yet been developed in EC. The mechanism of reported DNA demethylating agents in EC is illustrated in **Figure 3**.

At present, DNA demethylation reagents have been widely explored in hematological tumors and solid tumors, such as breast, liver, pancreatic and lung cancer, while studies in EC are limited to the preclinical stage and few in number. DNA demethylating agents still face great challenges in the treatment of EC, highlighting an urgent need to identify epigenetic drivers specific to EC and its subtypes. Treatment experience from other tumors suggests that not all patients benefit from demethylation monotherapy, underscoring the importance of actively seeking combination regiments. For instance, there are some superficial preclinical studies on the combination of DNMTi and histone deacetylase inhibitor (HDACi) in EC (128, 135, 136). Moreover, it is essential to note that demethylation agents are not region-specific and may reactivate oncogenes leading to therapy failure or even tumor progression. Therefore, novel demethylation reagents need to be developed that can, for example, bind to specific targets by linking unique sequences.

## 5 Conclusion and discussion

With the rapid advancement in tumor diagnosis and treatment, the significance of DNA methylation cannot be ignored. In this review, firstly, we comprehensively reviewed the biomarkers based on DNA methylation in EC, encompassing valuable research findings published over the past two years. These findings serve as a swift reference for researchers investigating specific genes and contribute to the development of mature methylation detection kits. Secondly, we delineated the multifaceted role of DNA methylation in risk assessment of EC, spanning carcinogenesis risk, feasibility of fertility preservation, and overall prognosis, tailoring personalized treatment plans for patients. For accurate interpretation of DNA methylation data in EC diagnosis and prognosis, large-scale multicenter studies conducted in a standardized manner are imperative for validation. Furthermore, we shed light on the current landscape of new drug development centered on DNA methylation. In contrast to hematological tumors, research on DNA demethylating agents in EC remains limited. Nonetheless, the existing theoretical framework and research outcomes tentatively hint at the feasibility of demethylating agents in EC treatment, necessitating further preclinical investigations to assess their efficacy and safety. The methylation regulation mechanism is intricate, and existing research mostly focuses on DNMTi. However, attention should also be directed towards methylation-binding proteins and demethylases as prospective targets for demethylation. Additionally, methylation may be involved in the occurrence of chemotherapy resistance, underscoring the importance of actively exploring combination therapy regimens to enhance efficacy and mitigate adverse events.

Admittedly, several limitations must be acknowledged to better interpret the findings of this review. One major challenge lies in the lack of consistency in data reporting across the cited studies. Key factors such as racial background, age, menopausal status, and tumor stage, which significantly influence DNA methylation patterns in EC, were inconsistently documented across studies, making it difficult to conduct comprehensive analyses or emphasize these variables. Moreover, methodological variability in DNA methylation detection platforms and analytical techniques, combined with the inclusion of some studies with small sample sizes, further complicates the comparability of results, limiting the generalizability of the synthesized findings. These limitations underscore the urgent need for more rigorous, standardized, and population-diverse studies to validate the role of DNA methylation in the diagnosis, risk assessment, and treatment of EC.

Overall, DNA methylation holds promising research prospects and expansive opportunities for exploration in EC, offering valuable insights for early diagnosis, risk assessment, and treatment. Nonetheless, overcoming the current limitations is essential for driving the clinical application of DNA methylation forward.
